# Use of whole genome sequencing of commensal *Escherichia coli* in pigs for antimicrobial resistance surveillance, United Kingdom, 2018

**DOI:** 10.2807/1560-7917.ES.2019.24.50.1900136

**Published:** 2019-12-12

**Authors:** Emma Stubberfield, Manal AbuOun, Ellie Sayers, Heather M O’Connor, Roderick M Card, Muna F Anjum

**Affiliations:** 1 Department of Bacteriology, Animal and Plant Health Agency, Weybridge, Surrey, United Kingdom; 2 University of East Anglia/Quadram Institute Bioscience, Norwich Research Park, Norwich, United Kingdom; 3 Department of Epidemiological Sciences, Animal and Plant Health Agency, Weybridge, Surrey, United Kingdom

**Keywords:** antimicrobial resistance, whole genome sequencing, genotype correlation, phenotype correlation

## Abstract

**Background:**

Surveillance of commensal *Escherichia coli*, a possible reservoir of antimicrobial resistance (AMR) genes, is important as they pose a risk to human and animal health. Most surveillance activities rely on phenotypic characterisation, but whole genome sequencing (WGS) presents an alternative.

**Aim:**

In this retrospective study, we tested 515 *E. coli* isolated from pigs to evaluate the use of WGS to predict resistance phenotype.

**Methods:**

Minimum inhibitory concentration (MIC) was determined for nine antimicrobials of clinical and veterinary importance. Deviation from wild-type, fully-susceptible MIC was assessed using European Committee on Antimicrobial Susceptibility Testing (EUCAST) epidemiological cut-off (ECOFF) values. Presence of AMR genes and mutations were determined using APHA SeqFinder. Statistical two-by-two table analysis and Cohen’s kappa (k) test were applied to assess genotype and phenotype concordance.

**Results:**

Overall, correlation of WGS with susceptibility to the nine antimicrobials was 98.9% for test specificity, and 97.5% for the positive predictive value of a test. The overall kappa score (k = 0.914) indicated AMR gene presence was highly predictive of reduced susceptibility and showed excellent correlation with MIC. However, there was variation for each antimicrobial; five showed excellent correlation; four very good and one moderate. Suggested ECOFF adjustments increased concordance between genotypic data and kappa values for four antimicrobials.

**Conclusion:**

WGS is a powerful tool for accurately predicting AMR that can be used for national surveillance purposes. Additionally, it can detect resistance genes from a wider panel of antimicrobials whose phenotypes are currently not monitored but may be of importance in the future.

## Introduction

*Escherichia coli* in animals comprise a diverse range of strains. They are a reservoir and indicator of antimicrobial resistance (AMR) genes that can be mobilised to other bacteria including zoonotic pathogens, and therefore pose a risk to human and animal health. Mobile elements such as plasmids and transposons are typically responsible for horizontal transfer of AMR genes [1,2]. Antimicrobial resistance can also be attributed to the expression of efflux pumps or single nucleotide polymorphisms (SNPs) present within chromosomal genes such as the DNA gyrase, that counteract the actions of certain antimicrobials [3].

Antimicrobial resistance is traditionally measured using phenotypic methods, for example, the minimal inhibitory concentration (MIC), and interpreted using breakpoints to determine resistance or susceptibility. The European Committee on Antimicrobial Susceptibility Testing (EUCAST) sets a clinical breakpoint that determines the likelihood of therapeutic success for treating infection; isolates with an MIC above this level are associated with a high level of therapeutic failure. EUCAST also defines an epidemiological cut-off (ECOFF) value which is used to differentiate the susceptible wild type bacterial population from non-wild type isolates with an acquired resistance mechanism. The European Union (EU) directive 2003/99/EC requests the harmonised monitoring and reporting of AMR, which is coordinated by the European Food Safety Authority (EFSA) and European Centre for Disease Prevention and Control (ECDC), to be carried out by EU countries for joint reporting of animal, food and human data [4]. The EU countries perform antimicrobial susceptibility testing on selected pathogenic and indicator organisms, which include *E. coli* [4,5], but no details are obtained on the underlying molecular mechanisms.

The presence of AMR genes harboured by bacteria can be determined by a variety of molecular methods, including PCR, DNA microarray and whole genome sequencing (WGS) [1]. Unlike PCR and microarrays, WGS offers the advantage of being able to screen the bacterial genome for multiple genes and mutations associated with AMR, which can be used to predict phenotypic susceptibility to antimicrobials [1,6] and to retrospectively detect newly identified AMR genes [7]. To identify AMR determinants, WGS data have to be screened against a database of AMR genes such as CARD or ResFinder [8,9].

In a 2017 report by EUCAST, it was stated that there is poor or non-existent evidence for using WGS as a method to infer antimicrobial susceptibility accurately [6]. Several recent studies have attempted to predict antimicrobial susceptibility from WGS data for a variety of bacteria, including *E. coli* [10-14]. When compared with phenotypic data, the predictions showed high concordance between genotype and phenotype with overall specificity and sensitivity > 95% [3]. However, many of these studies on *E. coli* are from clinical or known multidrug-resistant (MDR) isolates, and are limited to small panels of isolates (≤ 155).

We present an analysis on the association of gene presence from WGS with AMR phenotype in a large panel of commensal *E. coli* to predict their resistance to antimicrobials of human clinical and veterinary importance. We aimed to provide further evidence to support the use of WGS to enhance AMR surveillance and accurately predict antimicrobial susceptibility.

## Methods

### Bacterial isolates and antimicrobial susceptibility testing

In this retrospective study we compare phenotypic MIC values, interpreted using ECOFFs, with AMR genotypes in WGS data from 515 *E. coli* isolated from pooled caecal contents of healthy pigs collected at abattoir from 57 farms across the United Kingdom (UK) from 2014 to 2015 [15].

Isolates were selected on Brilliance UTI Agar (Oxoid, Basingstoke, UK) plates containing either 1 mg/L cefotaxime (CTX), 1 mg/L ciprofloxacin (CIP), no antibiotic (NoAB) and on Brilliance carbapenem-resistant Enterobacteriaceae (CRE) Agar (Oxoid). The British Society of Antimicrobial Chemotherapy (BSAC) agar dilution method [16] was used to test the susceptibility of each isolate against a panel of nine antimicrobials, of which two (apramycin and florfenicol) are only used in veterinary medicine. These antimicrobials span seven AMR classes of veterinary and/or human clinical relevance [17]: ampicillin (0.25–128 mg/L), apramycin (1–128 mg/L), cefotaxime (0.004–128 mg/L), ceftazidime (0.004–128 mg/L), ciprofloxacin (0.004–128 mg/L), florfenicol (0.25–128 mg/L), gentamicin (0.03–128 mg/L), sulfamethoxazole:trimethoprim 5:1 (0.15–640 mg/L for sulfamethoxazole and 0.03–128mg/L for trimethoprim) and tetracycline (0.25–128 mg/L). The MIC was defined as the lowest concentration that inhibited growth. Susceptibilities were interpreted using the EUCAST ECOFF values [18] because of a decision made by BSAC to migrate from BSAC to EUCAST methods was made after this study commenced [19]. For apramycin, there is no defined ECOFF value or clinical breakpoint for *E. coli* so the Danish Integrated Antimicrobial Resistance Monitoring and Research Programme (DANMAP)-proposed breakpoint of > 16 mg/L was used [20]. The definition of reduced susceptibility refers to isolates with an MIC above the ECOFF value and susceptible isolates refers to isolates with an MIC equal to or less than the ECOFF value.

### Whole genome sequencing

DNA was extracted and Illumina HiSeq 4000 System whole genome sequencing (WGS) (Illumina, San Diego, United States (US)) performed on the *E. coli* isolates and sequences were deposited in the European Nucleotide Archive (ENA) under study accession number PRJEB26317. The APHA SeqFinder pipeline was used to determine the presence of 2,044 AMR genes, including genes associated with the nine antimicrobials tested in this study [7]. Presence of AMR genes belonging to each AMR class considered was determined by mapping unassembled reads to a database of gene sequences, following quality control measurements described previously [7]. An AMR gene was considered present if there was 100% gene mapping to the reference in the APHA SeqFinder database of AMR genes, allowing up to 10 non-synonymous SNPs, with the exception of *floR* that was present at 99% gene mapping and *dfrA14*, *tet(A)* and *tet(M)* that were present at greater than 86% gene mapping. Genome assembles were generated using SPAdes version 3.7.0 [21] and AMR gene presence was corroborated using abricate (https://github.com/tseemann/abricate) [21]. To identify SNPs in chromosomal genes/regions, *gyrA*, *parC*, *parE* and *ampC* promoter, associated with resistance (cSNP-AMR), ClustalW gene alignments were performed in DNASTAR Lasergene 11 Core Suite (DNASTAR Inc, Madison, US) and these were then incorporated into the APHA SeqFinder pipeline. *E. coli* strain K12 MG1655 was used as the reference.

### Statistical analysis

The correlation between the presence/absence of AMR genes and/or associated SNPs from the WGS results and the susceptibility by MIC phenotypes was evaluated statistically by two-by-two table analysis, as performed previously for other phenotype/genotype correlations [22,23] where test specificity, sensitivity and the positive predictive value (PPV) and negative predictive value (NPV) of the test were calculated using the following criteria: (i) correlation between WGS-gene presence and MIC-resistant results as true positive (TP), (ii) WGS-negative and MIC-susceptible results as true negative (TN), (iii) WGS-gene presence but MIC-susceptible results as false positive (FP) and (iv) WGS-negative but MIC-resistant results as false negative (FN). The capability of the MIC and WGS for identifying AMR was compared using the Cohen’s kappa test (κ), which is a measure of agreement above that expected by chance, with a κ of 0 indicating that the test agrees as well as would be expected by chance, and a κ of 1 indicating complete agreement. When assessing the kappa test, a result of above 0.900 was interpreted as almost perfect, 0.800–0.900 as strong agreement, 0.600–0.790 as moderate agreement, 0.400–0.590 as weak agreement and 0.200–0.390 as minimal agreement [24].

## Results

### Minimum inhibitory concentration phenotype

Of 515 *E. coli* tested, only 56 isolates (11%) were susceptible to all antimicrobials tested, with the remaining isolates resistant to between one and seven antimicrobials. The most common reduced susceptibility was observed for tetracycline (77%, n = 395), ampicillin (57%, n = 295) and sulfamethoxazole:trimethoprim (57%, n = 291). The least common reduced susceptibility was detected for the two veterinary antimicrobials included, florfenicol (7%, n = 36) and apramycin (4.5%, n = 23).

### Antimicrobial resistance genotype

Seventy-eight of 515 isolates (15%) had no detectable AMR genes or cSNP-AMR present in our database, with the remaining isolates having between one and nine acquired AMR genes and/or cSNP-AMR associated with the antimicrobials tested. Approximately 55% (n = 283) of isolates harboured between three and seven AMR genes and/or cSNP-AMR showing a multidrug resistance genotype, with seven isolates harbouring genotypic resistances to all seven antimicrobial classes tested. The most common AMR genes included *bla_TEM-1b_* (45%), *tet*(A) (44%) and *tet*A(B) (36%) (Table 1), which corresponded with the high levels of reduced susceptibility to ampicillin and tetracycline detected by MIC .

**Table 1:** Antimicrobial resistance genes identified in *Escherichia coli* isolates from pooled caecal contents of healthy pigs, for nine antimicrobials, United Kingdom, 2018 (n = 515)

Antimicrobial class	Antimicrobial	AMR gene	Isolates positive for each gene (n)	Percentage of total (%)
**Aminoglycosides**	Apramycin^a^ and gentamicin	*aac3-Iva*	23	4.5
	Gentamicin	*ant2-Ia*	1	0.2
		*aac3-IId*	23	4.5
**β-lactamases**	Ceftazidime and cefotaxime (also ampicillin)	*bla_CMY-2_*	10	1.9
		*bla_CTX-M-1_*	9	1.8
		*bla_CTX-M-15_*	4	0.8
		*bla_SHV-12_*	5	1.0
		*ampC* promoter -42 C→T	18	3.5
	Ampicillin	*bla_TEM-1_*	6	1.2
		*bla_TEM-135_*	8	1.6
		*bla_TEM-30_*	5	1.0
		*bla_TEM-1b_*	230	44.7
		*bla_TEM-1c_*	10	1.9
		*bla_TEM-1d_*	2	0.4
**Phenicols**	Florfenicol^a^	*floR* ^b^	27	5.2
**Fluoroquinolones**	Ciprofloxacin	*qnrB5*	4	0.8
		*qnrB20*	1	0.2
		*qnrS1*	62	12.0
		*gyrA* Ser83Leu and Asp87Asn Ser83Leu Asp87Tyr Ser83Leu and Asp87Glu Asp87Ala Asp87Asn	161 80 60 11 8 1 1	31.3
		*parC* Ser80Lle Ser80Arg Ser80Arg and Glu84Lys	82 80 1 1	15.9
		*parE* Leu461Phe Ser458Ala	7 6 1	1.4
**Tetracyclines**	Tetracycline	*tet*A(B)	184	35.9
		*tet*(A)	217	43.9
		*tet*(C)	1	0.2
		*tet*(D)	1	0.2
		*tet*(M)	32	6.2
**Sulphonamides/** **trimethoprim**	Sulfamoxazole: trimethoprim (5:1)	*sul1*	69	13.4
		*sul2*	181	35.2
		*dfrA1*	66	12.8
		*dfrA12*	94	18.3
		*dfrA14* ^c^	39	7.6
		*dfrA15*	8	1.6
		*dfrA17*	53	10.2
		*dfrA21*	3	0.6
		*dfrA25*	1	0.2
		*dfrA5*	23	4.5
		*dfrA7*	2	0.4
		*dfrA8*	3	0.6

AMR: antimicrobial resistance.

^a^ Apramycin and florfenicol are only used in veterinary medicine.

^b^ *flo*R gene presence was determined at 99% mapping.

^c^ *dfr*A14, *tet*(A) and *tet*(M) gene presence was determined at greater than 86% mapping.

### Genotypic prediction of resistance phenotype

The relationship between genotype and phenotype was evaluated for the nine antimicrobials using the gene categories given in Table 1. Using the ECOFF value, overall correlation of WGS with MIC was 99% for test specificity and 98% for the tests’ PPV (Table 2). The kappa values for each antimicrobial tested ranged from 0.726 to 1.000, and predominantly showed strong agreement (κ > 0.800) between gene presence and reduced susceptibility (Table 2). The overall kappa score (κ = 0.914) indicated that WGS gene presence was highly predictive of reduced susceptibility and showed ‘almost perfect’ agreement with phenotypic MIC data from isolates. Test sensitivity was 91%, which although lower than test specificity, still showed strong agreement and could be because of the presence of yet unknown genes/mechanisms that were absent from our database.

**Table 2:** Correlation of whole genome sequencing and ECOFF or DANMAP values, test performances and kappa correlations for *Escherichia coli* isolates from pooled caecal contents of healthy pigs by antimicrobial, United Kingdom, 2018 (n = 515)

Antibiotic	Ciprofloxacin	Cefotaxime	Ceftazidime	Gentamycin	Florfenicol^a^	Ampicillin	Apramycin^a^	SXT	Tetracycline	Overall
Cut-off (mg/L)	ECOFF (> 0.06)	ECOFF (> 0.25)	ECOFF (> 0.5)	ECOFF (> 2)	ECOFF (> 16)	ECOFF (> 8)	DANMAP (> 16)	ECOFF (> 1)	ECOFF (> 8)	
**P+/G+**	218	45	45	45	27	283	23	258	387	1,330
**P-/G-**	249	464	441	468	479	218	492	209	114	3,135
**G+/P-**	10	1	1	0	0	2	0	15	6	34
**G-/P+**	38	5	28	2	9	12	0	33	8	136
**Test performances**										
**Specificity**	96.1%	99.8%	99.8%	100.0%	100.0%	99.1%	100.0%	93.3%	95.0%	98.9%
**Sensitivity**	85.2%	90.0%	61.6%	95.7%	75.0%	95.9%	100.0%	88.7%	98.0%	90.7%
**PPV**	95.6%	97.8%	97.8%	100.0%	100.0%	99.3%	100.0%	94.5%	98.5%	97.5%
**NPV**	86.8%	98.9%	94.0%	99.6%	98.2%	94.8%	100.0%	86.4%	93.4%	95.8%
**Kappa correlations**										
**Kappa**	0.814	0.9431	0.726	0.976	0.848	0.945	1.000	0.812	0.930	0.914

DANMAP: Danish Integrated Antimicrobial Resistance Monitoring and Research Programme; ECOFF: European Committee on Antimicrobial Susceptibility Testing epidemiological cut-off; G+: gene/SNP present; G-: gene/SNP absent; NPV: negative predictive value; P+: phenotype resistant; P-: phenotype sensitive; PPV: positive predictive value; SXT: sulfamethoxazole:trimethoprim.

^a^ Apramycin and florfenicol are only used in veterinary medicine.

Kappa correlation: almost perfect (> 0.900), strong agreement (0.800–0.900) and moderate agreement (0.600–0.790).

### Almost perfect agreement, kappa value > 0.900

**Figure 1:**
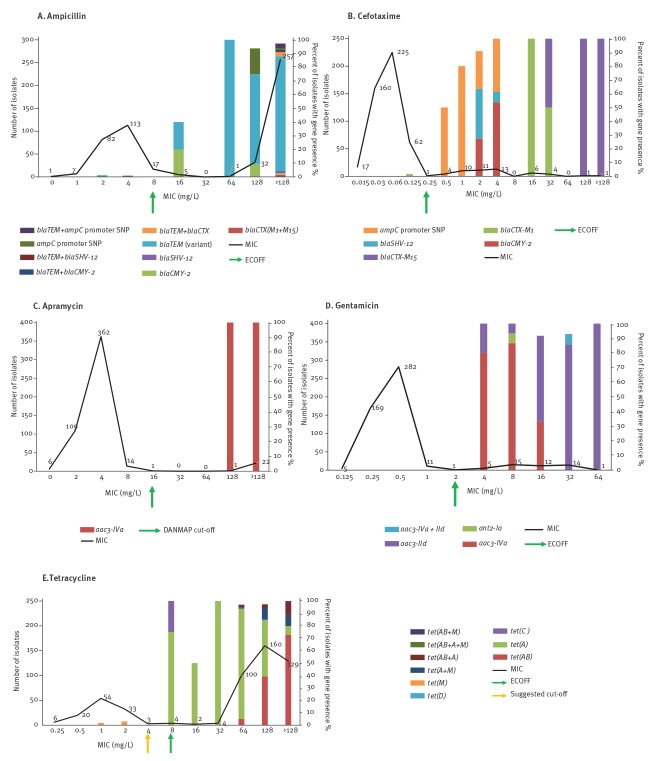
Almost perfect correlation between phenotype and genotype of *Escherichia coli* isolates from pooled caecal contents of healthy pigs by antimicrobial, United Kingdom, 2018 (n = 515)

ECOFF: European Committee on Antimicrobial Susceptibility Testing epidemiological cut-off; MIC: minimum inhibitory concentration.

Five antimicrobials (ampicillin, cefotaxime apramycin, gentamicin and tetracycline) showed almost perfect agreement (κ > 0.900) between phenotype and genotype. Fifty-seven percent of total *E. coli* isolates (n = 295) had reduced susceptibility to ampicillin, and 96% showed (n = 283) correlation with a resistance genotype; isolates harboured one or more β-lactamase resistance genes and/or a chromosomal *ampC* promoter mutation (Table 1, Figure 1A). Two isolates were phenotypically susceptible to ampicillin but harboured β-lactamase resistance genes; one isolate contained a *bla_CTX-M-1_* but was susceptible to ampicillin (MIC: 4 mg/L), ceftazidime (MIC: 0.5 mg/L) and cefotaxime (MIC: 0.125 mg/L), and the other ampicillin susceptible isolate (MIC: 2 mg/L) possessed two *bla_TEM_* variants (*bla_TEM-1b_* and *bla_TEM-30_*). For cefotaxime, 50 of 515 isolates (10%) had reduced susceptibility by ECOFF, of which, 45 isolates harboured a genetic resistance determinant present in our APHA SeqFinder database (Figure 1B). Twenty-seven isolates harboured transferable extended spectrum β-lactamase (ESBL) resistance genes (Table 1) and 18 contained the chromosomal *ampC* promoter mutation, which can lead to mutational de-repression or constitutive expression of AmpC [25]. The remaining five isolates showing reduced susceptibility (MIC: 0.5–2 mg/L), did not harbour an ESBL gene or chromosomal mutation in the *ampC* promoter.

The MIC distribution and the underlying genetic mechanisms present in isolates are shown.

Apramycin (C) is only used in veterinary medicine.

For apramycin, using the DANMAP proposed breakpoint, there was 100% agreement between the phenotype and genotype; 23 isolates showed reduced susceptibility (MIC: ≥ 128 mg/L) and harboured the *aac3*-Iva gene (Figure 1C). Similarly for gentamicin there was almost perfect correlation (κ = 0.976), where 45 of 47 isolates with reduced susceptibility harboured a gentamicin resistance gene (Figure 1D). A further two isolates with reduced susceptibility (MIC: ≥ 16 mg/L) did not harbour a gentamicin resistance gene; these isolates also showed reduced susceptibility to ampicillin (MIC: ≥ 128 mg/L), florfenicol (MIC: ≥ 64 mg/L) and sulfamethoxazole:trimethoprim (MIC: > 128 mg/L), but did not harbour the associated resistance genes, suggesting a possible alternative multidrug resistance mechanism, such as efflux.

Reduced susceptibility to tetracycline was the most common in our *E. coli* collection (77%, n = 395 isolates), and we were able to correlate this with a resistance genotype in 387 isolates which harboured a *tet* variant gene (Figure 1E). Six phenotypically susceptible isolates also harboured a *tet* variant, four of these were at the ECOFF value (MIC: 8 mg/L) and harboured either *tet*(A) or *tet*(C); two isolates with a MIC between 1 and 2 mg/L harboured *tet(*M), which is common in Gram-positive bacteria but has also been reported from *E. coli* and *Salmonella* [26,27].

### Strong agreement, kappa value 0.800 to 0.900

**Figure 2:**
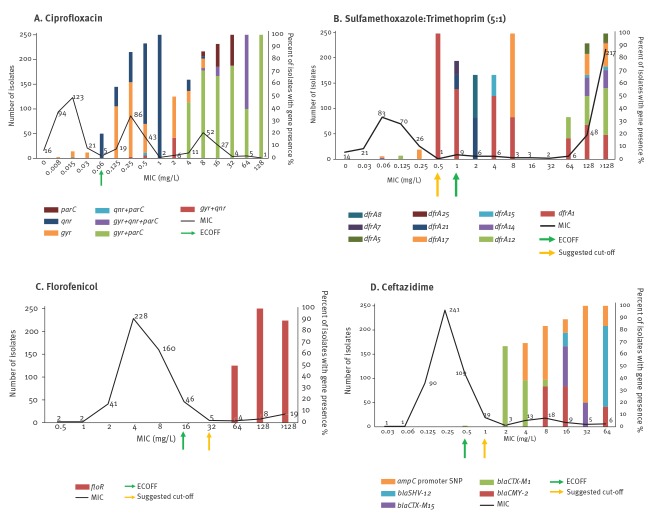
Strong and moderate correlation between phenotype and genotype of *Escherichia coli* isolates from pooled caecal contents of healthy pigs by antimicrobial, United Kingdom, 2018 (n = 515)

ECOFF: European Committee on Antimicrobial Susceptibility Testing epidemiological cut-off; MIC: minimum inhibitory concentration.

Three antimicrobials (ciprofloxacin, sulfamethoxazole:trimethoprim and florfenicol) showed very strong agreement (κ = 0.812 to 0.848) between the genotype and phenotype. Fifty percent (n = 256) of isolates showed reduced susceptibility to ciprofloxacin; 218 of these harboured a plasmid mediated quinolone resistance (PMQR) gene (26%, n = 57) and/or SNPs in the quinolone resistance determining regions (QRDR) of *gyrA* or *parC* (74%, n = 161). We noted that the MIC of isolates harbouring PMQR genes only (n = 57) was between 0.125 and 8mg/L, while those with QRDR mutations only (n = 152) ranged between 0.125 and 128mg/L. In the remaining 38 isolates with reduced susceptibility, no genotypic resistance mechanism was identified. Ten isolates were susceptible to ciprofloxacin but harboured genetic determinants; nine harboured mutations in the QRDR and one isolate harboured *qnrS1* and was at the ECOFF value (Figure 2A).

The MIC distribution and the underlying genetic mechanisms present in isolates are shown.

Florfenicol (C) is only used in veterinary medicine.

For sulfamethoxazole:trimethoprim, 57% isolates (n = 291) showed reduced susceptibility with 258 of these harbouring a *dfr* variant alone (n = 90) or in combination with a *sul* (n = 168). Two isolates had a *sul* variant alone (Figure 2B). For the remaining 31 isolates with reduced susceptibility to sulfamethoxazole:trimethoprim, no significant match with genetic determinants in our database were detected. As presence solely of *dfr* or in combination with *sul* resulted in reduced susceptibility; for phenotype and genotype correlation, these combinations were included and resulted in strong agreement (κ = 0.812). Fifteen susceptible isolates (5%) harboured a *dfr* gene alone (n = 10) or in combination with a *sul* (n = 5), seven of these were at the ECOFF (1 mg/L), and 37 susceptible isolates only harboured a *sul* variant.

Florfenicol is an antimicrobial solely used in veterinary medicine, and 36 isolates showed reduced susceptibility and resulted in strong agreement (κ = 0.848). The *floR* gene was present in 27 of these isolates with MIC > 32 mg/L, but in the remaining nine, no florfenicol resistance determinants were detected (Figure 2C). We noted that isolates harbouring *cml* or *catA1* genes, which are associated with chloramphenicol resistance, did not confer cross-resistance to florfenicol, typically showing MIC value < 32 mg/L (data not shown).

### Moderate agreement, kappa value 0.600 to 0.799

The lowest agreement was observed with ceftazidime (κ = 0.726); 73 isolates (14%) showed reduced susceptibility using ECOFF, but only 45 isolates harboured an ESBL gene or chromosomal *ampC* promoter mutation. The remaining 28 isolates did not harbour a resistance gene or chromosomal changes present in our database (Figure 2D).

### Effect of adjusting the epidemiological cut-off

For florfenicol, others [28,29] have suggested a ECOFF value for *E. coli* isolates tested from swine and cattle of > 32 mg/L as an alternative to the current one at > 16 mg/L. When the higher MIC cut-off value was applied to our dataset only 31 of 515 (6%) isolates showed reduced phenotypic susceptibility, and reduced the number of false negative isolates to four. The higher cut-off value also improved sensitivity and raised kappa correlation for florfenicol to the almost perfect correlation category, from 0.848 to 0.927. A similar scenario was observed for tetracycline where lowering cut-off value from > 8mg/L to > 4 mg/L, (Figure 1E) increased the kappa correlation from 0.930 to 0.945 so only two isolates were false positive. For eight isolates, the correlation did not change; they remained as false negative.

For sulfamethoxazole:trimethoprim, the MICs were determined using a 5:1 ratio that is relevant for veterinary medicine [30], however, the ECOFF value is based on a 19:1 ratio as used in human clinical settings. Based on the ratio of 5:1 sulfamethoxazole:trimethoprim used, we suggest that a lower ECOFF value of > 0.5 mg/L is more appropriate for the methodology applied here and will need to be tested for wider application (Figure 2B). However, its application increased the number of isolates with reduced susceptibility from 291 to 300 (58%), with 210 of these isolates containing a *dfr* variant. As a result, the number of susceptible isolates harbouring a *dfr* gene halved, improving the kappa agreement between MIC phenotype and WGS gene presence from 0.812 to 0.836. For ceftazidime, comparison of the MIC with WGS at a slightly higher cut-off of > 1 mg/L (Figure 2D) improved the kappa correlation, from 0.726 to 0.889, because of a decrease in the number of false negative isolates.

Using the ECOFF cut-off values, the number of isolates that showed complete phenotype/genotype concordance for all nine antimicrobials in their susceptibility profile was 394 (77%). For the remaining 121 isolates, there were discrepancies (false positive or false negative results) in up to five antimicrobials. Using the adjusted cut-off values for the four antimicrobials above, the numbers of isolates with genotype/phenotype concordance for all nine antimicrobials across their susceptibility profile increased to 419 (81%), with the number of discrepant isolates decreasing to 96.

## Discussion

In this study, we explored the use of WGS data as an alternative to the traditional phenotypic method, MIC, used to determine AMR. At the time of this study, the BSAC agar dilution MIC was the accepted method for determining susceptibility, and in 2016, BSAC migrated to the EUCAST broth microdilution method in order to harmonise resistance prediction interpretations across Europe [19]. For this reason, EUCAST ECOFF values were applied to our data. A future study comparing the genotype results with phenotypes derived from broth microdilution would be of interest. However, as there was almost perfect agreement between WGS and MIC for the majority of antimicrobials when ECOFF values were used with agar dilutions, the improvements may or may not be substantial when using broth microdilutions. Additionally, there were no considerable changes to the kappa values obtained in this study when isolate WGS data were tested with other AMR pipelines available (data not shown).

Sulfamethoxazole:trimethoprim, florfenicol, ceftazidime and ciprofloxacin, which had the poorest agreement, nevertheless showed strong to moderate kappa values. However, when the ECOFF value was adjusted for several aforementioned antimicrobials, following analysis of the MIC distribution with gene presence, we noted an improvement in the kappa correlation, i.e. an increase in the number of isolates that were true positives and true negatives. The majority of discrepancies in our results were because of false negative correlations, i.e. isolates with reduced susceptibility lacking a relevant genetic resistance determinant from our database, suggesting that unknown resistance genes may be present in these bacteria. However, the overall WGS/MIC specificity of 99%, the predictive value of a positive test being 98% and a kappa value of 0.930 was encouraging, and adds to the growing number of studies recommending the use of AMR genotyping [10,12-14].

In other studies where *E. coli* WGS was compared with phenotype [10,12,13], reported discrepancies mainly included phenotypically-susceptible isolates harbouring a resistance gene. In this study, a small number of such false positive isolates (14/31) had an MIC at or below the ECOFF value; this was most commonly observed for sulfamethoxazole:trimethoprim and ciprofloxacin. In the case of sulfamethoxazole:trimethoprim, determining the phenotype from genotype was complicated by the different mechanisms of resistance, including the expression of efflux pumps and cell wall permeability [31].

This study focused on known acquired resistance determinants. However, there are a number of other chromosomal genes where SNPs and variations in expression could result in resistance, e.g. *folA*-encoding dihydrofolate reductase for trimethoprim [11], or expression of multidrug efflux systems such as the *mar* operon [32,33], that were not investigated and could explain some of the false negative results that were obtained. Also, there may be improvement in phenotypic and genotypic correlations if an ECOFF was attained appropriate for the 5:1 ratio used in veterinary medicine. Other limitations of the WGS approach are that only known genes can be determined [1,6] and that it does not take genes that may be present but not expressed or the effect of multiple resistance genes present for the same class of antimicrobial into account [34].

However, WGS can be used to identify the presence of novel-acquired mechanisms to explain discrepancies, for example the identification of the pleuromutilin resistance gene *tva*(A) in *Brachyspira* spp. [35]. New resistance mechanisms can be added to the AMR database and the WGS can be screened again to perform retrospective analysis once new/novel genes are detected, as shown following *mcr* detection in 2015 [36]. Therefore, isolates found to have moderate kappa correlations in this study may be harbouring novel genes/mechanisms that require further investigation.

The EUCAST report on the role of WGS in AMR susceptibility testing of bacteria recommended that the primary MIC comparator for WGS predictions be the ECOFF, but it encouraged using the clinical breakpoints as a secondary comparator, acknowledging that doing so would be more challenging with our current knowledge [6]. Although our study found almost perfect correlation (κ > 0.9) between phenotype/genotype for most of the antimicrobials considered using ECOFF cut-offs, clinical breakpoints available for four antimicrobials showed much lower correlation (Supplementary Table S1). Clinical breakpoints are based on the success of treatment and therefore factors other than the MIC distribution alone are taken into consideration, e.g. host, clinical treatment data and pharmacokinetics of the drug [37]; properties that cannot be predicated from bacterial genome sequence alone.

WGS also provides the potential to predict susceptibility to a wider range of antimicrobials, including those not routinely tested in MIC panels. The APHA SeqFinder pipeline screens for 2,044 genes. In addition to the genes associated with the nine antimicrobials tested in this study (Table 1), a further 29 genes with predicted reduced susceptibility to other aminoglycosides (*aac*6-IId, *aad*(various), *ant*3–1a, *aph*3, *aph4*, *str*A and *strB* genes), chloramphenicol (*cml, cat*), streptothricin (*sat*2) and macrolides (*erm*B, *inu*F, *mef*B and *mph*A and *mphB*) were also detected in the 515 *E. coli* isolates. WGS data can also be used to provide further information on strains, including the multilocus sequence types, phylogeny, plasmids and plasmid types [36] that are important in assessing transmission of AMR. However, standardisation of WGS methodologies and analysis of the data is required before this technology is applied in AMR reference laboratories for routine surveillance activities.

In conclusion, the results of this study demonstrate that the use of WGS, a technological advancement over traditional phenotyping, should be considered an alternative way to monitor antimicrobial resistance in bacteria by national and pan-European surveillance programmes.

## Supplementary Data

526000 Supplementary Material
