# Supplementary material for: Use of whole genome sequencing of commensal Escherichia coli in pigs for antimicrobial resistance surveillance, United Kingdom, 2018
Source: Euro Surveill. 2019 Dec 12;24(50):1900136. doi: 10.2807/1560-7917.ES.2019.24.50.1900136 (PMC6918588; doi:10.2807/1560-7917.ES.2019.24.50.1900136)
Supplement: Supplementary Material [file 19-00136_SupplementaryMaterial.pdf]

Supplementary Material

This supplementary material is hosted by *Eurosurveillance* as supporting information alongside the article "Use of whole genome sequencing of commensal *Escherichia coli* in pigs for antimicrobial resistance surveillance, UK, 2018", on behalf of the authors, who remain responsible for the accuracy and appropriateness of the content. The same standards for ethics, copyright, attributions and permissions as for the article apply. Supplements are not edited by *Eurosurveillance* and the journal is not responsible for the maintenance of any links or email addresses provided therein.

Table S1: Statistical analysis using two-by-two tables of WGS and clinical breakpoints and Kappa correlations. Kappa correlation: >0.900 = almost perfect; 0.800-0.900 = strong agreement and 0.600-0.790 = moderate agreement. Abbreviations: P+ = phenotype resistant, P- = phenotype sensitive, G+ = gene/SNP present, G- = gene/SNP absent, PPV = positive predictive value, NPV = negative predictive value.

**Antibiotic**	**Ciprofloxacin**	**Cefotaxime**	**Ceftazidime**	**Gentamycin**	**Overall**
**cut-off (mg/L)**	**Clinical** 0.5	**Clinical** 2	**Clinical** 4	**Clinical** 4	**Clinical**
**P+/G+**	92	25	34	40	191
**P-/G-**	271	470	465	468	1674
**G+/P-**	136	20	12	5	173
**G-/P+**	16	0	4	2	22
**specificity**	67%	96%	97%	99%	91%
**sensitivity**	85%	100%	89%	95%	90%
**PPV**	40%	56%	74%	89%	52%

**NPV**	94%	100%	99%	100%	99%
**Kappa**	0.3587	0.6953	0.7928	0.9121	0.6063
